# Calycosin Promotes Angiogenesis Involving Estrogen Receptor and Mitogen-Activated Protein Kinase (MAPK) Signaling Pathway in Zebrafish and HUVEC

**DOI:** 10.1371/journal.pone.0011822

**Published:** 2010-07-29

**Authors:** Jing Yan Tang, Shang Li, Zhen Hua Li, Zai Jun Zhang, Guang Hu, Lorita Chi Veng Cheang, Deepa Alex, Maggie Pui Man Hoi, Yiu Wa Kwan, Shun Wan Chan, George Pak Heng Leung, Simon Ming Yuen Lee

**Affiliations:** 1 Institute of Chinese Medical Sciences, University of Macau, Macao, China; 2 School of Biomedical Sciences, Faculty of Medicine, The Chinese University of Hong Kong, Hong Kong, China; 3 State Key Laboratory of Chinese Medicine and Molecular Pharmacology, Department of Applied Biology and Chemical Technology, The Hong Kong Polytechnic University, Hong Kong, China; 4 Department of Pharmacology and Pharmacy, Faculty of Medicine, The University of Hong Kong, Hong Kong, China; Universidade Federal do Rio de Janeiro (UFRJ), Brazil

## Abstract

**Background:**

Angiogenesis plays an important role in a wide range of physiological processes, and many diseases are associated with the dysregulation of angiogenesis. *Radix Astragali* is a Chinese medicinal herb commonly used for treating cardiovascular disorders and has been shown to possess angiogenic effect in previous studies but its active constituent and underlying mechanism remain unclear. The present study investigates the angiogenic effects of calycosin, a major isoflavonoid isolated from *Radix Astragali*, *in vitro* and *in vivo*.

**Methodology:**

*Tg(fli1:EGFP)* and *Tg(fli1:nEGFP)* transgenic zebrafish embryos were treated with different concentrations of calycosin (10, 30, 100 µM) from 72 hpf to 96 hpf prior morphological observation and angiogenesis phenotypes assessment. Zebrafish embryos were exposed to calycosin (10, 100 µM) from 72 hpf to 78 hpf before gene-expression analysis. The effects of VEGFR tyrosine kinase inhibitor on calycosin-induced angiogenesis were studied using 72 hpf *Tg(fli1:EGFP)* and *Tg(fli1:nEGFP)* zebrafish embryos. The pro-angiogenic effects of calycosin were compared with raloxifene and tamoxifen in 72 hpf *Tg(fli1:EGFP)* zebrafish embryos. The binding affinities of calycosin to estrogen receptors (ERs) were evaluated by cell-free and cell-based estrogen receptor binding assays. Human umbilical vein endothelial cell cultures (HUVEC) were pretreated with different concentrations of calycosin (3, 10, 30, 100 µM) for 48 h then tested for cell viability and tube formation. The role of MAPK signaling in calycosin-induced angiogenesis was evaluated using western blotting.

**Conclusion:**

Calycosin was shown to induce angiogenesis in human umbilical vein endothelial cell cultures (HUVEC) *in vitro* and zebrafish embryos *in vivo* via the up-regulation of vascular endothelial growth factor (VEGF), VEGFR1 and VEGFR2 mRNA expression. It was demonstrated that calycosin acted similar to other selective estrogen receptor modulators (SERMs), such as raloxifene and tamoxifen, by displaying selective potency and affinity to estrogen receptors ERα and ERβ. Our results further indicated that calycosin promotes angiogenesis via activation of MAPK with the involvement of ERK1/2 and ER. Together, this study revealed, for the first time, that calycosin acts as a selective estrogen receptor modulator (SERM) to promote angiogenesis, at least in part through VEGF-VEGFR2 and MAPK signaling pathways.

## Introduction

Angiogenesis is the establishment of the mature blood vessel network through expansion and remodeling of the pre-existing vascular primordium. Blood vessel formation through angiogenesis involves the induction of new sprouts, coordinated and directed endothelial cell migration, proliferation, sprout fusion (anastomosis) and lumen formation [1]. It is a process tightly regulated by a variety of pro-angiogenic factors such as the estrogen receptors (ERs). ERs are a group of transcriptional factors that belong to the nuclear receptor superfamily and are activated by estrogen. In addition to its reproductive function, ER also plays an important role in the cardiovascular system [2]. Previous studies have demonstrated that ER expressed in endothelial cells mediates angiogenesis through both classical genomic, and rapid non-genomic, mechanisms [3], [4], [5]. Ligands of ER such as 17β-estradiol (E2), estradiol and raloxifene have been shown to induce endothelial cells proliferation and migration [6], [7]. Meanwhile, some isoflavonoids possessing estrogenic properties that are regarded as selective estrogen receptor modulators (SERMs), also provide cardiovascular benefits, including regulation of endothelial cells proliferation, differentiation, adhesion, migration and kinase activation through interacting with ER [8], [9].

Natural products, such as certain Chinese medicines, contain a variety of angiogenic compounds. It has been demonstrated that Rg1 and Rb1, the two prevalent saponins of *Ginseng*, have opposing effects in modulating angiogenesis [10]. Another Chinese medicine *Radix Astragali*, which is rich in isoflavonids, is often used either as a single herb or in combination with other Chinese medicines as formula for treating myocarditis [11], heart failure [12], myocardial infarction [13], pulmonary hypertension [14], [15], [16], [17], chronic hepatitis [18], diabetes [19], [20] and systemic lupus erythematosus [21] among others. *Danggui buxue tang* (DBT), a Chinese herbal concoction composed of *Radix Astragali* and *Angelica sinensis*, is commonly prescribed to treat menopausal irregularity and menstrual disorders [22], [23], [24]. DBT triggered specific phosphorylations of ERα and ERK1/2 in the cultured human breast cancer cell line, MCF-7 [25].

Despite *Radix Astragali* have been shown to stimulate angiogenesis in some studies, the mechanism underlying its angiogenic activity remains unclear [26]. The major bioactive constituents of *Radix Astragali* are saponins and flavonoids, including astragaloside (I∼VIII), calycosin, formononetin, ononin and their glucosides [27], [28]. Among these isoflavonoids, calycosin is the candidate with most potential to develop as a small-molecule angiogenic agent, due to its benefits upon endothelial cells [29]. Calycosin protects HUVECs from hypoxia-induced barrier impairment by increasing intracellular energetic sources and promoting regeneration of cAMP levels, as well as improving cytoskeleton remodeling. Our previous study illustrated that *Radix Astragali* extract (RAE) possesses pro-angiogenic effects upon human umbilical vein endothelial cells (HUVECs), which involve the VEGF-VEGFR2 and PI3K-Akt-eNOS pathways [30]. HPLC chromatography revealed that the compositions of formononetin, calycosin, (6aR, 11aR)-9,10-dimethoxy-3-hydroxypterocarpan and saponins (astragaloside I, II and IV) in the RAE were 8.15%, 0.77%, 0.01% and 0.88% of the whole extract, respectively. In regards to the preliminary screening of the angiogenic effects of these constituents, calycosin was found to be the most potent pro-angiogenic agent among all. This present study examines whether calycosin acts on ER and promotes angiogenesis in HUVEC cultures *in vitro* and a transgenic zebrafish model *in vivo*.

## Results

### Pro-angiogenic effect of calycosin in zebrafish

**Figure 1:**
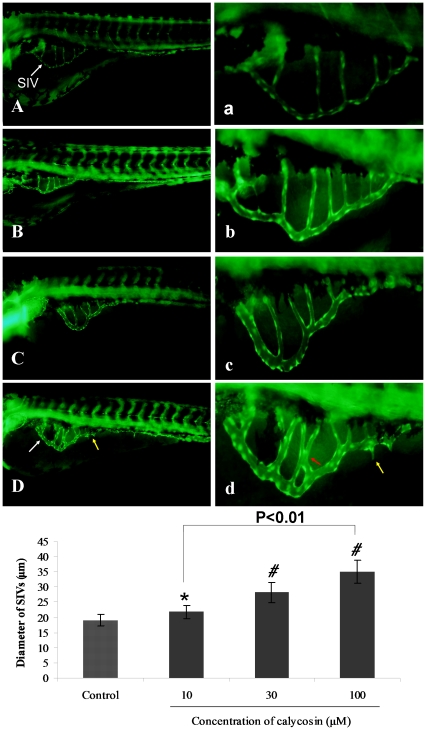
The effects of calycosin treatment on blood vessel formation in SIVs of *Tg(fli1:EGFP)* zebrafish embryos. (A) Control: embryo treated with 0.1% DMSO at 96 hpf, SIVs appear as a smooth basket-like structure. (B–D) Calycosin: embryo treated with 10, 30, 100 µM calycosin at 72 hpf for 24 h, leads to enlarged SIV basket stretching into the posterior yolk extension. (a–d) Enlarged SIV region (×4.5) of A–D respectively. White arrows indicating the enlarged vessels, yellow and red arrows indicate sprouting and intersectioning branches respectively. (E) Calycosin increases SIV diameter in a dose-dependent manner. Data are plotted as mean±SEM, (n = 3), *P<0.05, *#*P<0.001.

In zebrafish, angiogenic vessel development does not begin until 20 hpf (hours-post fertilization), and changes in subintestinal vein vessels (SIVs) are detected after 72 hpf. Fig. 1A shows that the SIVs of *Tg(fli1:EGFP)* zebrafish line treated with 0.1% DMSO at 96 hpf developed as a smooth basket-like structure. Following calycosin treatment (10, 30, 100 µM) from 72 hpf to 96 hpf, the diameter of SIVs increased in a dose-dependent manner (Fig. 1B–D). Quantitative analysis confirmed a significant (P<0.05 and P<0.001) dose-dependent effect of calycosin on diameter of SIVs compared with the control group (Fig. 1E).

**Figure 2:**
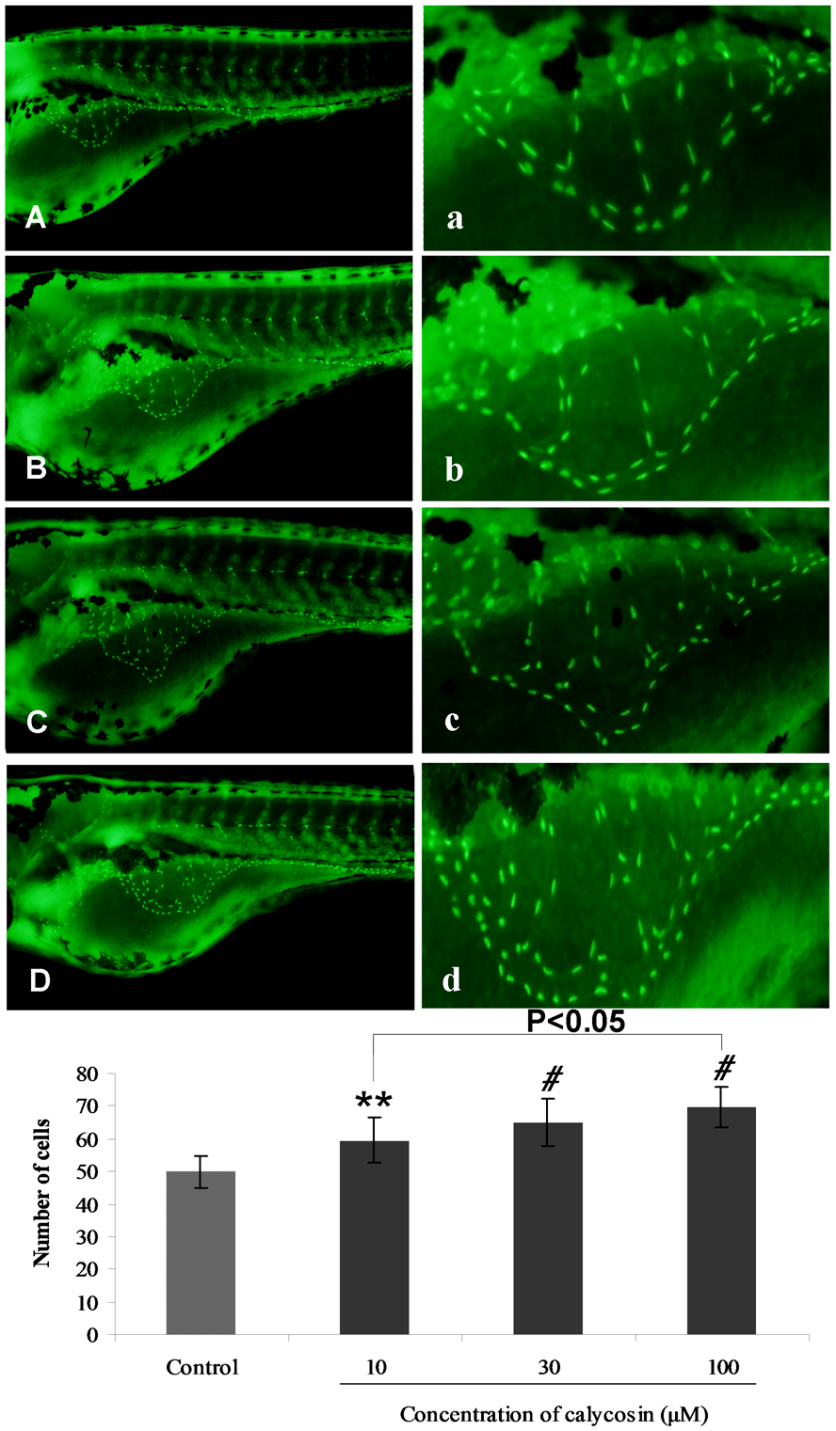
The effects of calycosin on endothelial cells population in SIVs of *Tg(fli1:nEGFP)* zebrafish embryos. Each green light point represents one endothelial cell (GFP+). (A) Control: embryo treated with 0.1% DMSO at 96 hpf. (B–D) Calycosin: embryo treated with 10, 30, 100 µM calycosin at 72 hpf for 24 h, leads to an increase in endothelial cells. (a–d) Enlarged SIV region (×4.5) of A–D respectively. (E) Calycosin increases the number of endothelial cells in the SIV region in a dose-dependent manner. Data are plotted as mean±SEM, (n = 3), **P<0.01, *#*P<0.001.

In order to determine whether the change of blood vessel phenotype (Fig. 1B–D) involves merely a transient vasodilation effect, or genomic action on stimulating endothelial cells proliferation, *Tg(fli1:nEGFP)* zebrafish embryos were used to demonstrate the angiogenic effect of calycosin. *Tg(fli1:nEGFP)* fish were engineered similarly to *Tg(fli1:EGFP)* except that *Tg(fli1:nEGFP)* harbor nuclear-localized GFP expression, permitting real-time *in vivo* analysis of individual endothelial cells [31]. These results show that calycosin treated (10, 30, 100 µM) SIVs contained significantly (P<0.01 and P<0.001) more endothelial cells (Fig. 2B–D) throughout the SIV region than the control group (Fig. 2A). Quantitative analysis indicates that calycosin induced an approximately 1.5 times increase in endothelial cells population compared with the control (Fig 2E).

### Detection of mRNA expression in calycosin treated zebrafish

In order to identify molecular targets of the angiogenic effects of calycosin in zebrafish, mRNAs from different groups were isolated and reverse transcribed to cDNA, and relative gene expression determined using real-time PCR. VEGFA is a fundamental mediator of physiological and pathophysiological angiogenesis [32], and acts through tyrosine kinase receptors. VEGFR2 (fetal liver kinase, also known as KDR and Flk-1) has a higher affinity for VEGF and is a major transducer of the VEGF signal in endothelial cells [33], [34].

**Figure 3:**
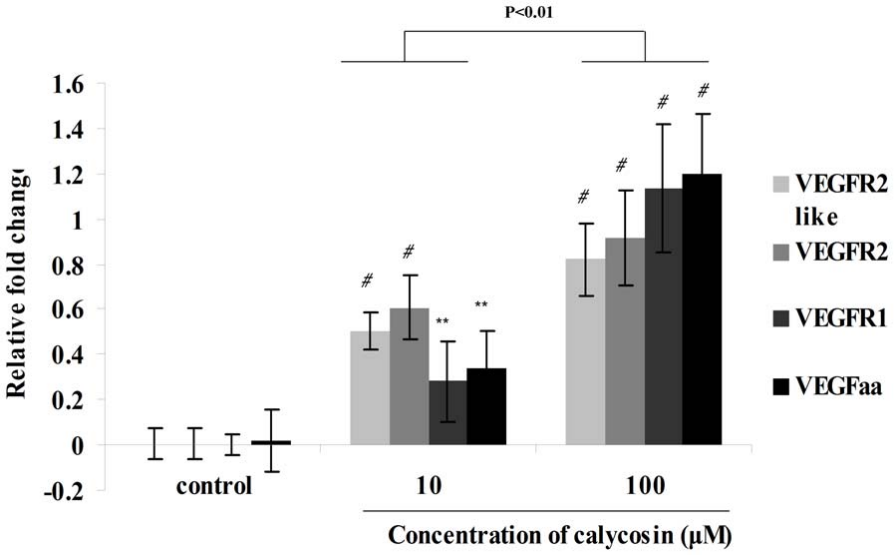
Gene expression of calycosin treated zebrafish. Data are expressed as mean ±SEM from three individual experiments. **P<0.01, *#*P<0.001 vs control group; P<0.01 vs calycosin group.

The bar charts in Fig. 3 represent the gene expression of VEGFA after treatment with 100 µM calycosin for 6 h. There was an increase trend of mRNA expression level compared to the control (1.2-fold at 100 µM), and calycosin caused a significant increase in mRNA expression of VEGFR1 (1.1-fold at 100 µM; P<0.001), Flk1A (0.8-fold at 100 µM; P<0.001) and Flk1B (0.9-fold at 100 µM; P<0.001). Hence, these results suggest that the up-regulation of expression of these genes caused by calycosin could contribute to the pro-angiogenic effects of calycosin observed in zebrafish.

### VEGFRs are important in calycosin-induced angiogenic effects

**Figure 4:**
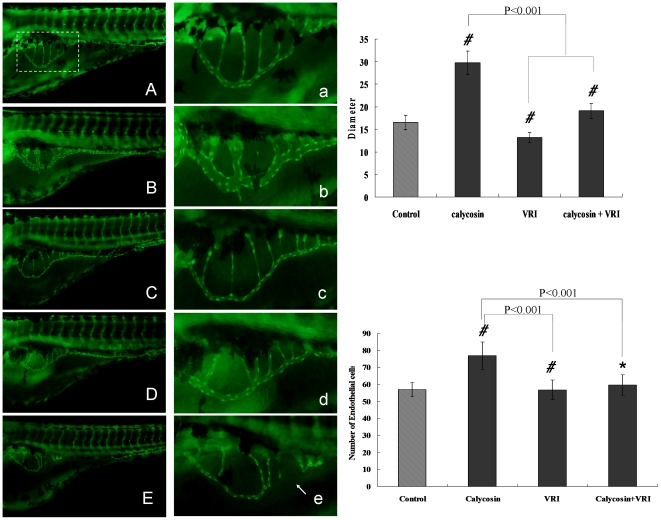
The effects of VEGFR tyrosine kinase inhibitor on calycosin-induced angiogenesis in zebrafish embryos. (A) Control: embryo treated with 0.1% DMSO at 96 hpf. (B) Calycosin: embryo treated with calycosin (100 µM) at 72 hpf for 24 h. (C & E) VRI: embryo treated with low concentration (100 ng/ml, C) and high concentration (1 µg/ml, E) of VRI at 72 hpf for 24 h. (D) VRI and calycosin: embryo treated with both VRI (100 ng/ml) and calycosin (100 µM) at 72 hpf for 24 h. (a–e) Enlarged SIV region (×4.5) of A–E respectively. (F) Effects of calycosin and/or VRI on the diameter of SIV compared with the control group. Data are plotted as mean±SEM, (n = 3), *#*P<0.0001. (G) Effects of calycosin and/or VRI on the number of endothelial cells in SIV region compared with the control group. Data are plotted as mean±SEM, (n = 3), *P<0.05, *#*P<0.001.

VEGFR tyrosine kinase inhibitor II (VTKI, VRI), a pyridinyl-anthranilamide compound that displays both antiangiogenic and antitumor properties, has been shown to potently inhibit the kinase activities of VEGFR1 and VEGFR2 [35]. We found that VRI, when in high concentration (1 µg/ml), caused significant (P<0.001) defects in angiogenesis in zebrafish embryonic development (Fig. 4E). Indeed, a lower concentration of VRI (100 ng/ml), which itself had no effect (Fig. 4C), caused significant (P<0.001) defects in calycosin-induced angiogenesis in zebrafish embryonic development (Fig. 4D). Quantitative analysis confirmed that a low concentration of VRI (100 ng/ml) was sufficient to reverse the calycosin-induced angiogenic effects to control levels (Fig. 4F & 4G). This indicates that, in exerting its effect, calycosin interacts with VEGF receptors (VEGFRs), further confirming that calycosin-induced angiogenesis, at least in part, involves the VEGF- VEGFR2 signaling pathway.

### Calycosin acts directly but differentially with ERα and ERβ

**Figure 5:**
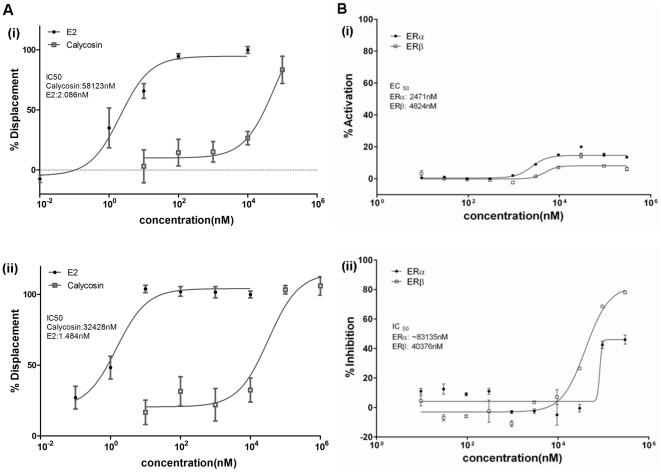
Cell-free and cell-based estrogenic assays. (A) Dose-response curves for competitive binding assay. Calycosin and 17-β-estradiol (E2) at the concentrations shown competitively bind with (i) ERα and (ii) ERβ, which caused displacement of Fluormone™ ES2 from ER; (B) Dose-response curves for GeneBLAzer β-lactamase reporter-gene assay. (i) Agonistic activities and (ii) antagonistic activities of calycosin at ERα and ERβ were determined in ERα-UAS-*bla* GripTite™ and ERβ-UAS-*bla* GripTite™ cell lines respectively. Results are presented as mean±SEM. (n≥2 independent experiments), P<0.01 between different ER subtypes followed by two-way ANOVA.

Since ERs are potential targets of calycosin [25], its binding affinities to ERα and ERβ were evaluated by fluorescent polarization competitive binding assay. 17-β-estradiol (E2), a native agonist for both ERα and ERβ, was used as a positive control. In this study, E2 displayed strong binding affinity for ERα and ERβ (ERα: IC_50_ = 2.086 nM, Fig. 5A–i; ERβ: IC_50_ = 1.484 nM, Fig. 5A–ii). Calycosin displaced Fluormone™ ES2 and bound to ERα and ERβ in a dose-dependent manner (Fig. 5A–i & Fig. 5A–ii). The binding affinities of calycosin to ERα and ERβ were not as strong as that of E2, with an IC_50_ value approximately 10^4^-fold higher than that of E2 and its lower maximum displacement. On the other hand, the IC_50_ value of calycosin at ERα (IC_50_ = 58.123 µM, Fig. 5A–i) was very similar to that at ERβ (IC_50_ = 32.428 µM, Fig. 5A–ii).

To further examine the transcriptional agonistic/antagonistic action of calycosin on ERs, GeneBLAzer β-lactamase reporter-gene experiments were performed. Calycosin showed weak agonistic activities at both ERα and ERβ (maximum activity was 14.6% and 8.6%, respectively, Fig. 5B–i). In contrast, the antagonistic activities of calycosin against E2 at ERα and ERβ were significant (maximum inhibition was 46% and 82%, respectively; P<0.01; Fig. 5B–ii). Thus, our results suggest that calycosin is a partial agonist/antagonist for both ERα and ERβ.

Calycosin also displayed receptor-selective potency and efficacy in the reporter gene assay. In the agonist activity assay, calycosin showed ERα selectivity with a 2-fold reduction in EC_50_ value and a 2-fold increase in maximal activation compared with ERβ (Fig. 5B–i). However, calycosin was more potent and efficacious at ERβ than at ERα in the antagonist activity assay, showing a 2-fold reduction in IC_50_ value and a 2-fold increase in maximal inhibition (Fig. 5B–ii).

Comparison of angiogenic effects of calycosin with other classical SERMs in zebrafish embryos.

**Figure 6:**
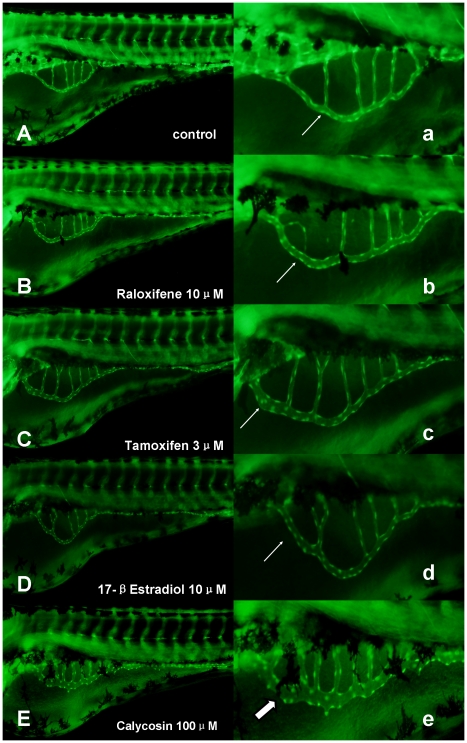
The effects of calycosin, raloxifene and tamoxifen in SIVs of *Tg(fli1:EGFP)*. (A) Controls: were treated with 0.1% DMSO at 96 hpf, showing no effect on vessel formation (B–E) were treated with 10 µM raloxifene, 3 µM tamoxifen, 10 µM 17-β-Estradiol and 100 µM calycosin at 72 hpf for 24 h. (a–e) Enlarged SIV region (×4.5) of A–E respectively. Abnormal phenotype of blood vessel formation in SIVs was indicated by white arrow, showing slight increase in vessel diameter. Significant increase in vessel diameter was indicated by thick white arrow.

Raloxifene is a SERM approved for clinical use in osteoporosis, and has been suggested to induce cardioprotection in women at high risk of coronary heart disease [36]. Another example of a SERM is tamoxifen, which is an antagonist of the estrogen receptor and is used in treating breast cancer [37]. E2 represents the major estrogen in humans, which modulates various vascular functions, including inflammation, wound healing, and angiogenesis [38], [39], [40]. As shown in Fig. 6, only calycosin exhibited a significant angiogenic effect in SIVs (Fig. 6e, thick arrow), while no obvious changes were observed in the raloxifene (10 µM), tamoxifen (3 µM) and 17-βEstradiol (10 µM) groups (Fig. 6b–d, arrows) at their highest non-toxic doses in zebrafish embryos.

### Calycosin promotes angiogenesis in HUVEC *in vitro*

**Figure 7:**
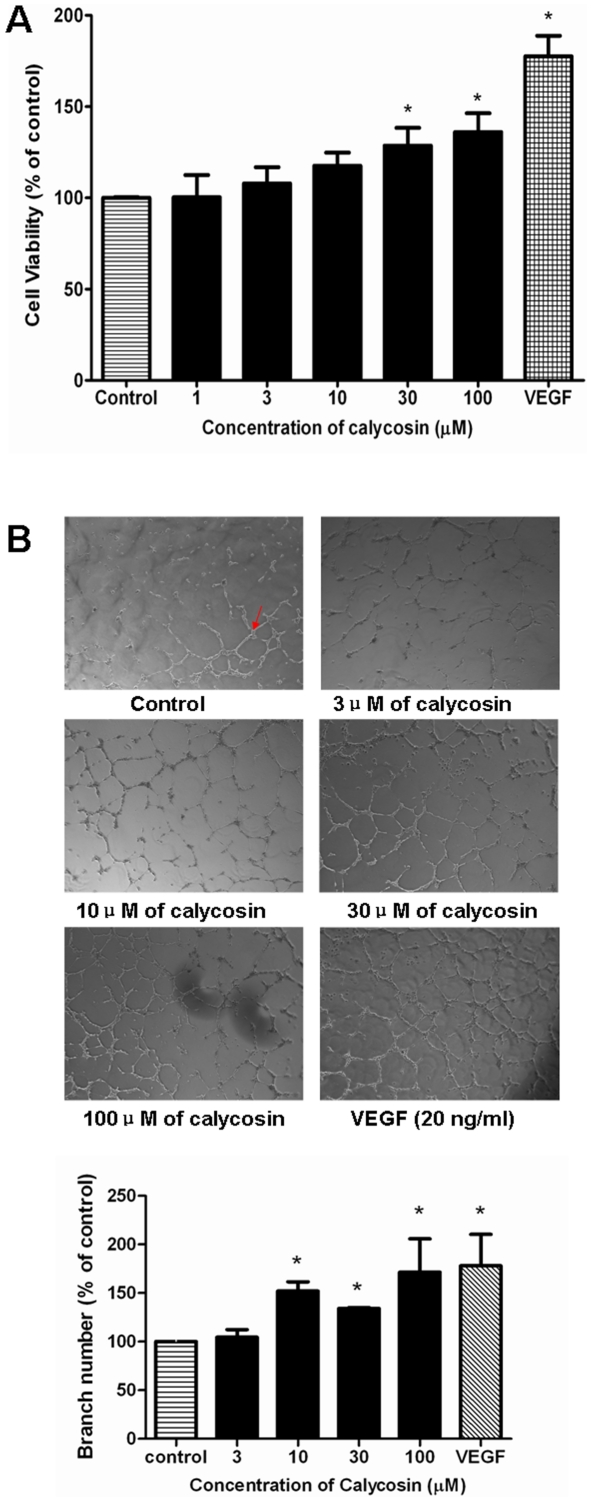
The effects of calycosin on HUVECs *in vitro*. (A) Effects of calycosin on proliferation of HUVEC by XTT assay. HUVECs were seeded in 96-well plates and incubated with calycosin at different concentrations. Cell proliferation was assessed using XTT assay; (B) Tube formation of calycosin-treated HUVECs on Matrigel. HUVECs cultured on 3-dimensional Matrigel in treatment of calycosin (3 µM, 10 µM, 30 µM and 100 µM). Cells receiving 0.1% DMSO served as vehicle control. Number of branching points in different concentrations of calycosin-treated HUVECs was calculated by computer software (Metamorph). Results are expressed as percentage of control (100%) in mean±SEM (n≥3 independent experiments), *P<0.05 versus control.

The effect of calycosin on HUVEC proliferation was evaluated using an XTT assay. Following a 24 h starvation, HUVECs were cultured in low serum medium supplemented with calycosin (1 µM–100 µM; 48 h). Cell viability was estimated by determining the amount of formazon product formed in the cell culture medium. As shown in Fig. 7A, calycosin promoted cell proliferation in a dose-dependent manner. The maximum increase of cell viability induced by calycosin was 36% at 100 µM, compared to vehicle control. A significant (P<0.05) increase in cell proliferation was also observed in VEGF-treated cells (77%), which served as the positive control.

The process of angiogenesis is complex, and typically consists of proliferation and alignment to form tubular structures [41]. To test the ability of calycosin to induce HUVEC capillary tube formation, a Matrigel model was used. When HUVECs were cultured on Matrigel – a solid gel of mouse basement membrane proteins – cells aligned easily and formed hollow, tube-like structures. Fig. 7B shows that a very low level of tube formation was observed when HUVECs were plated on Matrigel in low-serum medium, whereas morphological changes were observed after treatment with calycosin. Quantitative analysis indicates that calycosin stimulated HUVECs to form more branching points (Fig. 7B). The number of branching points increased in a dose-dependent manner and reached its maximum (71%) at a calycosin concentration of 100 µM. A significant (P<0.05) increase in branching points was also observed in VEGF-treated cells (71%), which served as the positive control.

### Calycosin induces angiogenesis via activation of MAPK signaling pathway

ERK1/2, one of the major targets of the MAPK signaling pathway, has been implicated in the regulation of angiogenesis for different functions including cell proliferation, migration and survival [41], [42]. To evaluate the rapid activation of these kinases, western blotting was used to examine the phosphorylation of ERK1/2 following calycosin treatment.

**Figure 8:**
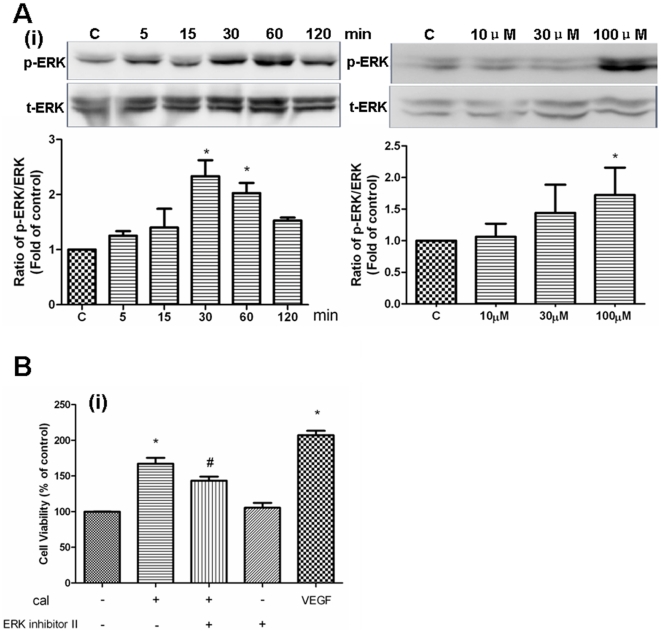
Role of MAPK signaling in calycosin-induced angiogenesis. (A) Effects of calycosin on ERK1/2 activation. HUVEC were incubated with calycosin (100 µM) at indicated time or with calycosin in different concentrations for 30 min. Expressions of phospho-ERK1/2 and total-ERK1/2 were analyzed by western blotting and quantified by densitometry. The values indicate the relative densitometric units. Results are represented as mean±SEM (n = 3 independent experiments), * P<0.05 versus control. (B) Effect of ERK activation inhibitor peptide II on calycosin-induced HUVEC proliferation. HUVECs were pre-treated with 0.5 µM ERK activation inhibitor peptide II (ERK inhibitor II) for 1 h before the addition of calycosin (100 µM). Changes in HUVEC proliferation were determined 48 h later by XTT assay. 20 ng/ml VEGF was used as the positive control in this experiment. “cal” is the abbreviation of calycosin. Results are expressed as percentage of vehicle control (100%) in mean±SEM (n≥3 independent experiments), *P<0.05 versus vehicle control, # P<0.05 versus calycosin.

Firstly, phospho-ERK1/2 and total-ERK1/2 were detected following treatment with calycosin after different time durations. Calycosin stimulated the phosphorylation of ERK1/2 in a time-dependent manner (Fig. 8A–i), which reached a plateau at 30–60 min, and rapidly declined thereafter. However, the total protein levels of ERK1/2 remained unaffected throughout the course of these experiments. Furthermore, phosphorylation of ERK1/2 in HUVECs was enhanced in a dose-dependent manner after incubating with different concentrations of calycosin (Fig. 8A–i). The phosphorylation of ERK1/2 reached its maximum at a calycosin concentration of 100 µM, consistent with the results of the XTT assay. These data demonstrate that calycosin stimulated rapid activation of ERK1/2 in a time- and dose-dependent manner.

To further confirm the involvement of ERK1/2 in calycosin-mediated angiogenesis, a specific blocker was applied to examine its effect on calycosin-induced proliferation. HUVEC proliferation was significantly (P<0.05) increased after incubating with calycosin, but this was significantly (P<0.05) inhibited after pre-treatment with ERK activation inhibitor peptide II (Fig. 8B–i). Altogether, these results indicate that ERK1/2-dependent pathways are involved in calycosin-induced HUVEC proliferation.

### Calycosin induces HUVEC proliferation via interaction with ER

**Figure 9:**
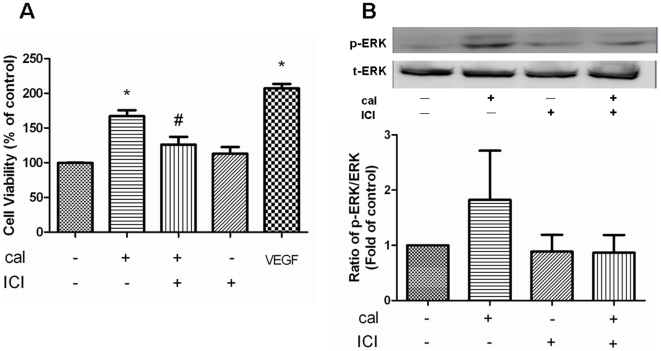
Role of ER in calycosin-induced angiogenesis. (A) Effects of ICI182, 780 on calycosin-induced HUVEC proliferation. HUVECs were pre-treated with ICI182, 780 (30 µM) before the addition of calycosin (100 µM). Data are expressed as percentage of vehicle control (100%) in mean±SEM (n = 3 independent experiments), *P<0.05 versus control, #P<0.05 versus calycosin. (B) Effect of ICI182, 780 on calycosin-induced activation of ERK1/2. Calycosin-stimulated phosphorylation of ERK1/2 was completely reversed by the absence of ICI182, 780 (30 µM). Expression of phospho-ERK1/2 and total-ERK1/2 was analyzed by western blotting and quantified by densitometry. The values indicate the relative densitometric units of the p-ERK1/2 bands with the density of the control band set arbitrarily at 1.0. Results are represented as mean±SEM. “cal” and “ICI” are the abbreviations of calycosin and ICI182, 780 respectively.

To confirm whether ER is involved in the angiogenic activity of calycosin, the effects of ER inhibitors on calycosin-induced HUVEC proliferation, and ERK1/2 activation, were examined. Fig. 9A demonstrates that calycosin significantly promoted the HUVEC proliferation by 67% (P<0.05), while the ER inhibitor (IVI182, 780) significantly reduced the proliferation by 40% (P<0.05). Western blotting revealed that expression of phospho-ERK1/2 was markedly enhanced in calycosin-treated HUVECs, whereas ICI182, 780 (30 µM) suppressed phosphorylation of ERK1/2 to control levels (Fig. 9B). Total ERK1/2 protein levels were unaffected by these treatments. Altogether, these results show that the effects of calycosin on HUVEC proliferation and ERK1/2 activation could be reversed by ER inhibition.

## Discussion

Proliferation of endothelial cells is a key process in angiogenesis [43]. The present study demonstrates that calycosin enhances endothelial cells proliferation in HUVECs *in vitro*, and in zebrafish embryos *in vivo*. Both blood vessel diameter and number of endothelial cells increased following calycosin treatment of transgenic zebrafish. Thus, these findings suggest that calycosin possesses pro-angiogenic activity.

Furthermore, these results show that the calycosin-induced phenotypic change in zebrafish involved activation of angiogenesis-related signaling pathways. Changes in mRNA expression levels of several angiogenesis-specific markers were determined. VEGF, also known as vascular permeability factor (VPF), was originally described as an EC-specific mitogen, a potent angiogenic factor [44], as well as an essential growth factor for vascular ECs. Formation of new blood vessels is orchestrated by a plenitude of different proteins, including cell adhesion molecules, ECM components and VEGFRs. Gene targeting experiments have provided insights into the functions of VEGFRs [45], [46]. Although inactivation of each individual VEGFR can cause embryonic lethality at mid-gestation, they have different functions [47], [48]. VEGFR2 is the receptor that initiates the main signaling pathways activated by VEGF. The main function of VEGFR1 appears to be in regulating binding between VEGF and VEGFR2 [49]. In this investigation, the results of real-time PCR illustrate that calycosin extract increased VEGF expression, as well as having a tendency to upregulate expression of VEGFR1 and VEGFR2. Moreover, VKRI, an inhibitor of VEGFR1 and VEGFR2, was shown to potently inhibit the kinase activities of these two proteins [35]. These data confirmed the predominant involvement of these angiogenesis-specific targets in calycosin-induced increases in endothelial cell number and blood vessel diameter at SIVs in zebrafish, and further supported the hypothesis that these clear phenotypic changes were as a result of angiogenesis stimulation.

Menopausal women suffer from many health problems such as hot flushes, sweating and mood swings; they are also more prone to cardiovascular disease, bone density reduction and osteoporosis. These problems are mainly due to deficiencies of ovarian hormones, especially estrogen. Therefore, hormone replacement therapy (HRT) is often applied to relieve such menopausal symptoms, and offer protection against osteoporosis and cardiovascular diseases [50]. However, recent epidemiological studies, and randomized trials, have revealed that women who used HRT had an increased risk of developing breast cancer, strokes and thromboembolisms [50], [51]. These reports contributed to the development of SERM, which is defined as molecules binding with ER and producing a change in the biological activities of the receptor with cell, or tissue, specificity.

Cell-free and cell-based estrogenic assays both revealed that calycosin competitively bound with ERα and ERβ. In addition, calycosin also displayed selective potency and affinity to ERα and ERβ in reporter-gene assays. Clinical and animal studies have suggested multiple benefits of SERM, and several SERMs have already been clinically approved, including raloxifene and tamoxifen. Recent findings have demonstrated the beneficial effects of these two classical SERMs upon the vascular system [52], [53], [54], [55]. Since raloxifene and tamoxifen share the same/similar antagonistic action with calycosin at ERβ, we compared the angiogenic effects of the three compounds in zebrafish embryos. Of the three, only calycosin promoted significant angiogenic development in the SIVs of zebrafish embryos.

A previous study investigated the activities of compounds demonstrated to be active in zebrafish embryo bioassays, in both zebrafish and mammalian cell lines [56]. Interestingly, only half of the 14 compounds were shown to be active in both embryos and either one of the cell lines, revealing that they exerted direct action upon cells. In our results, calycosin not only promoted angiogenesis in zebrafish but also enhanced endothelial cells proliferation and tube formation in HUVECs *in vitro*, both of which are standard tests for angiogenesis. Although no study has been carried out to identify bioequivalent doses between cell cultures and zebrafish, our results suggest that calycosin, at least in part, exerts direct action upon endothelial cells. Thus, we can further investigate the mechanism of action of calycosin in cell culture.

Many studies have shown that MAPK signaling pathway activation plays a vital role in the proliferation, migration and morphogenesis of endothelial cells induced by pro-angiogenic factors [57], [58]. To further elucidate the mechanism of the angiogenic activity of calycosin, activation of MAPK signaling was detected. It was shown that calycosin stimulated ERK1/2 activation rapidly in HUVECs (Fig. 8A). In addition, an ERK1/2-specific inhibitor effectively reversed calycosin-induced HUVEC proliferation (Fig. 8B). Thus, these results indicate that calycosin promotes angiogenesis via activation of MAPK with the involvement of ERK1/2.

Since calycosin selectively modulates ER transcriptional activation, as well as promoting angiogenesis, to further elucidate the relationship between these two activities, the effects of ER inhibitor ICI182, 780 on calycosin-induced HUVEC proliferation and the expression of phospho-ERK1/2 were examined. *In vitro* and *in vivo* studies have demonstrated that estrogen and ER agonists promote angiogenesis in endothelial cells via ERs [3], [59]. It has also been shown that inhibition of ER reduces angiogenesis induced by an ER agonist [5]. Here, we showed that ICI182, 780 significantly (P<0.05) decreased calycosin-induced HUVEC proliferation (Fig. 9A). Moreover, recent studies indicate that 17-β-estradiol stimulates ERK1/2 phosphorylation through ERα activation in endothelial cells [60]. In this sense, our data revealed that calycosin-stimulated ERK1/2 activation was also abrogated by ER inhibition (Fig. 9B). Altogether, our data suggest that calycosin stimulates activation of ER and MAPK signaling pathways, which may contribute to the pro-angiogenic activity of calycosin.

In conclusion, this present study provides evidence that calycosin from *Radix Astragali* acts as a novel SERM, since calycosin was shown to competitively bind with ERα and ERβ, as well as selectively modulate ER transcriptional activities. We also show that calycosin treatment promotes several features of angiogenesis in HUVECs *in vitro*. Our studies elucidate the mechanism of the angiogenic activity of calycosin on HUVEC cells, where it promotes angiogenesis through activation of ER and the MAPK signaling pathway to play multiple roles in regulating cell proliferation and morphogenesis. Finally, our findings provide inspiration for further development of *Radix Astragali* and calycosin as therapeutic agents for the treatment of problems associated with estrogen deficiency, such as cardiovascular diseases in post-menopausal women.

## Materials and Methods

### Ethics Statement

All animal experiments were conducted according to the ethical guidelines of ICMS, University of Macau and the protocol was approved by ICMS, University of Macau.

### Chemicals and reagents

Kaighn's modification of Ham's F12 medium (F-12K), fetal bovine serum (FBS), phosphate-buffered saline (PBS), charcoal-stripped fetal bovine serum (CS-FBS), penicillin-streptomycin (PS), 0.25% (w/v) trypsin/1 mM EDTA and nitric oxide indicators DAF-FM diacetate were all purchased from Invitrogen (Carlsbad, CA, USA). Endothelial cell growth supplement (ECGS), heparin, gelatin, ER antagonist ICI182, 780, 17β-estradiol, Raloxifene hydrochloride, Tamoxifen, SNP and wortannin were supplied by Sigma (St Louis, MO). Growth factor reduced (GFR) Matrigel™ basement membrane matrix was obtained from BD Biosciences (Bedford, MA). ERK activation inhibitor peptide II was obtained from Biocalchem (Darmstadt, Germany). Vascular endothelial growth factors (VEGF) were obtained from R&D Systems (Minneapolis, MN). Anti-p-ERK1/2 antibody, anti-ERK1/2 antibody and goat anti-rabbit IgG HRP-conjugated antibody were all purchased from Cell Signaling Technology (Berverly, MA). Dimethyl sulfoxide (DMSO) was acquired from SIGMA and the calycosin(≥99%) was extracted at our laboratory. Calycosin was dissolved in DMSO to form a 100 mM solution. VEGFR tyrosine kinase inhibitor II (VTKI) was purchased from Calbiochem Company/EMD Chemicals Inc (Cat. No. 676481) and was dissolved in DMSO to form a 1 mg/ml solution.

### Maintenance of zebrafish and its embryos

EGFP is expressed in all endothelial cells and each nucleus of *Tg(fli-1:EGFP)* and *Tg(fli-1:nEGFP)* zebrafish embryos. All types of zebrafish were maintained as described in the Zebrafish Handbook [61].

### Embryo collection and drug treatment

Zebrafish embryos were generated by natural pair-wise mating (3–12 months old) and were raised at 28.5°C in embyro water. Healthy, hatched zebrafish were picked out at 3 dpf and distributed into a 12-well microplate with 10 to 15 fish in each well. Different concentrations (10, 30, 100 µM) of calycosin, raloxifene or tamoxifen solutions were then added to wells and incubated at 28°C for 24 h. Embryos receiving DMSO (0.1%) served as vehicle controls and were equivalent to no treatment. Each experiment was repeated at least three times, with 30 embryos per group. VTKI was dissolved in DMSO as stock. Zebrafish embryos, *Tg(fli-1:EGFP)* and *Tg(fli-1:nEGFP)*, were treated with inhibitor dissolved in embryo water from 3 dpf at the concentration indicated, controlled by DMSO treated embryos. Embryos were maintained using standard methods.

### Morphological observation of zebrafish

At 96 hpf, zebrafish were removed from microplates and observed for viability and gross morphological changes under a fluorescence microscope (Olympus IX81 Motorized Inverted Microscope, Japan) equipped with a digital camera (DP controller, Soft Imaging System, Olympus). Images were analyzed with Axiovision 4.2 and Adobe Photoshop 7.0.

### Assessment of vascular changes

Three random points in SIVs of *Tg(fli1:EGFP)* zebrafish embryos were chosen for vessel diameter measurement using AxiovisionLE 4.1. Numbers of endothelial cells in SIVs of *Tg(fli1:nEGFP)* zebrafish embryos were assessed by direct counting of the total number of green light points. Each green light point represents one endothelial cell (GFP+).

### Total RNA extraction, reverse transcription, and real-time PCR

Zebrafish embryos at 72 hpf were treated with calycosin for 6 h. Total RNA was extracted from 30 zebrafish embryos of each treatment group using the RNeasy Mini Kit (Qiagen, USA) in accordance with the manufacturer's instructions. RNA was reverse transcribed to single-strand cDNA using SuperScript™ III First-Strand Synthesis System for RT–PCR (Invitrogen™, USA), followed by real-time PCR using the TaqMan® Universal PCR Master Mix and 250 nM custom TaqMan primers for zebrafish Flk1A, Flk1B, VEGFR1, VEGFA2 (Applied Biosystems, USA) in the ABI 7500 Real-Time PCR System (Applied Biosystems). The expression of Flk1A, VEGFA2 mRNA was normalized to the amount of *bactin1*, using the relative quantification method described by the manufacturer.

The zebrafish *bactin1* primers were 5′-CAAGATTCCATACCCAGGAAGGA-3′ (F) and 5′-CAAGATTCCATACCCAGGAAGGA-3′(R) (Applied Biosystems, USA).

The zebrafish Flk1A (kdrl) primers were 5′- GACCATAAAACAAGTGAGGCAGAAG-3′ (F) and 5′- CTCCTGGTTTGACAGAGCGATA-3′(R) (Applied Biosystems, USA).

The zebrafish Flk1B (kdr) primers were 5′- CAAGTAACTCGTTTTCTCAACCTAAGC-3′ (F) and 5′-GGTCTGCTACACAACGCATTATAAC-3′(R) (Applied Biosystems, USA).

The zebrafish FLT1 primers were 5′-AACTCACAGACCAGTGAACAAGATC-3′ (F) and 5′-GCCCTGTAACGTGTGCACTAAA-3′(R) (Applied Biosystems, USA).

The zebrafish VEGFA2 primers were 5′-GATGTGATTCCCTTCATGGATGTGT-3′ (F) and 5′-GGATACTCCTGGATGATGTCTACCA-3′ (R) (Applied Biosystems, USA).

### HUVEC culture

Human umbilical vein endothelial cells (ATCC, Manassas) were cultured in F-12K medium with 2 mM L-glutamine, 1.5 g/l sodium bicarbonate, 100 µg/ml heparin, 30 µg/ml endothelial cell growth supplement and 10% FBS at 37°C in a humidified atmosphere of 5% CO_2_. Tissue culture flasks, 96-well plates and 6-well plates were pre-coated with 0.1% gelatin. Cells were exposed to culture medium with 10% CS-FBS instead of normal FBS for at least 1 day before experiments. Cultures were then starved with low-serum medium (contain 0.5% CS-FBS) for either 24 h in cell proliferation assays, or overnight in other assays. All assays were conducted using low cell passage cells (2–5 passages).

### ER fluorescence polarization competitive binding assay

The binding affinity of calycosin to ER-α and β was evaluated by the commercially available competitor assay (Invitrogen, Carlasbad, CA) according to the manufacturer's instructions. In brief, ER was added to fluorescently tagged ER ligand (Fluormone™ ES2) to form ER/Fluormone™ ES2 complexes with a high fluorescent polarization value. Displacement of fluorescently tagged ligands by unlabeled ligands decreased fluorescent polarization, resulting in a low value. In this system, changes in intensity of polarization reflect displacement of fluorescently tagged ligands. In 96-well plates, serial dilution of calycosin (1 nM to 3×10^5^ nM) or the ER agonist 17-β-estradiol (10^4^ nM to 10^−2^ nM) were added to ER/Fluormone™ ES2 complexes to compete with ES2 for binding to ER. Plates were incubated at room temperature for 2 h and fluorescent polarization values measured using a Multilabel Counter (Perkin Elmer, Singapore). Results were expressed as percentages of maximum displacement induced by 17-β-estradiol (10 µM).

### Cell-based ER transcriptional response by GeneBLAzer β-lactamase reporter-gene assay

Assays were preformed by Invitrogen (USA) as described in literature [62]. Briefly, GeneBLAzer β-lactamase reporter-gene assays were performed to measure the agonistic or antagonistic activities of calycosin at ER. For ER agonist activity assay, 4 µl of a 10×serial dilution of 17-β-estratiol served as the control agonist (starting concentration 10 µM, 3-fold dilute manner), or calycosin (starting concentration 300 µM, 3-fold dilute manner), was added to the appropriate wells of a 384-well plate. 32 µl of cell suspension and 4 µl of Assay Media were added to each well to bring the final volume to 40 µl. Plates were incubated for 16–24 h, then 8 µl of 1 µM substrate loading solution was added to each well, and plates incubated for another 2 h at room temperature. For the antagonist activity assay, cells were grown and prepared as above. 4 µl of a 10×serial dilution of 4-hydroxytamoxifen (starting concentration 100 nM) for ERα, RU-496 (starting concentration 10 µM) for ERβ or calycosin (starting concentration 300 µM, 3-fold dilute manner) was added to cells. Cells were pre-incubated with calycosin and the antagonist control for 30–60 min, then 4 µl of 17-β-estradiol was added to wells at the pre-determined EC_80_ concentration. Plates were then incubated for another 16–24 h. 8 µl of 1 µM substrate loading solution was added to each well, and plates incubated for 2 h at room temperature. All results were measured using a fluorescence plate reader. Results of agonist activity assays were expressed as percentage activation of the defined maximum activation induced by 17-β-estradiol (10 µM). For the antagonist activity assay, inhibition responses were expressed as percentage inhibition in the presence of EC_80_ concentration of 17-β-estradiol according to the previous agonist activity assay.

### HUVEC viability by XTT assay

HUVECs were trypsinised and seeded at 10^4^ cells/well in 96-well gelatin coated plates. After 24 h, complete medium was removed and renewed with hormone-free low serum (0.5% CS-FBS) medium, and samples incubated for 24 h in order to starve HUVECs to achieve a quiescent state. After these pre-incubations, different concentrations (1 µM-100 µM) of calycosin medium were replaced. Cells receiving DMSO (0.1%) served as vehicle controls, and were equivalent to no treatment. For inhibition assays, HUVECs were pretreated with inhibitors (10 µM ICI182, 780 and 1 µM ERK activation inhibitor peptide II) for 60 min before addition of calycosin (100 µM). Cells receiving DMSO (0.1%) served as vehicle control and were equivalent to no treatment. On the other hand, cells cultured in VEGF (20 ng/ml) served as positive controls. After 48 h, cell proliferation was assessed by XTT for 4 h. The spectrophotometrical absorbance of each well was measured by a Multilabel counter (Perkin Elmer, Singapore). The wavelength used to measure absorbance of the formazan product was 490 nm and the reference wavelength was 690 nm. Cell viability data were expressed as percentage of cell viability calculated.

### Tube formation assay on HUVEC

The effect of calycosin on HUVEC differentiation was examined by *in vitro* tube formation on Matrigel [63]. Confluent HUVECs were harvested and diluted (9×10^4^ cells) in 500 µl low serum medium containing 3–100 µM calycosin, which were then seeded on 1∶1 Matrigel (v/v) coated 24-well plates in triplicate at 37°C for 7 h. Cells receiving DMSO (0.1%) served as vehicle controls, and were equivalent to no treatment. Besides, cells cultured in 20 ng/ml VEGF served as positive controls (data not shown). The network-like structures were examined under an inverted microscope (at 50× magnification). The tube-like structures were defined as endothelial cord formations that were connected at both ends. The number of branching points in three random fields per well was quantified by Metamorph Imaging Series software.

### Western blotting analysis

Cells were treated with 100 µM calycosin for different time durations (5–120 min) in the time course study. 20 ng/ml VEGF was used as a positive control while medium with 0.1% DMSO served as a negative control. To observe dose-dependent effects of calycosin, 10 µM, 30 µM and 100 µM calycosin were used to treat HUVECs for 30 min in culture medium. For inhibition assays, HUVECs were pretreated with 10 µM ICI182, 780 for 60 min prior to the addition of 100 µM calycosin. Cells were then washed with PBS and lysed for 30 min on ice with lysis buffer (0.5 M NaCl, 50 mM Tris, 1 mM EDTA, 0.05% SDS, 0.5% Triton X-100, 1 mM PMSF, pH 7.4). Cell lysates were centrifuged at 11000×g for 20 min at 4°C. Protein concentrations in the supernatants were measured using the bicinchoninic acid assay (Pierce, Rockford, IL). Supernatants were electrophoresed on 12% SDS-PAGE, and transferred to polyvinylidene diuoride (PVDF) membranes, which were then blocked with 5% non-fat milk. Immunoblot analysis was undertaken by incubation with anti-p-ERK1/2 antibody and anti-ERK1/2 antibody at 4°C overnight. After washing, membranes were incubated for 1 h at room temperature with horseradish peroxidase-conjugated goat anti-rabbit IgG. Proteins were detected using an advanced enhanced ECL system (GE Healthcare, Little Chalfont, Buckinghamshire, UK). Semi-quantifications were performed with densitometric analysis by Quantity One software.

### Statistical analysis

Data was analyzed with unpaired two-tailed Student's t-tests or one-way ANOVA followed by Tukey's multiple comparison test, using GraphPad Prism 5.0 software (San Diego, CA). Curve fitting was carried out using GraphPad Prism 5.0 (nonlinear fit, variable slope sigmoidal dose-response model). Data were expressed as mean±SEM from individual experiments. Differences were considered as significant at P<0.05.
